# The Heating of Milk as It Affects the Nutrition and Health of the Infant

**Published:** 1909-03

**Authors:** J. M. Fortescue-Brickdale

**Affiliations:** Assistant Physician, Bristol Royal Infirmary


### THE HEATING OF MILK AS IT AFFECTS THE NUTRITION AND HEALTH OF THE INFANT.

BY

J. M. FORTESCUE-BRICKDALE, M.A., M.D.,
*Assistant Physician, Bristol Royal Infirmary.*

[ABSTRACT.]

THIS paper deals with the alterations in the physico-chemical character of milk produced by heat, not with the effect on bacteria or their products. These alterations may be described in three groups. The first group consists of the obvious changes, namely the changes in taste and colour (due to action of heat on lactose), and the formation of a " skin " (due to drying of protein on the surface, and not peculiar to milk). These changes are not of great importance as regards the infant. The next group consists of changes not appreciable by the ordinary observers, namely the precipitation of calcium, and alterations in the organic phosphorus compounds. The former renders the caseinogen less coagulable *in vitro*, but it is not certain whether the same effect is produced *in vivo*. It appears, however, that heated milk is less easily acted on by pepsin and trypsin, and in all probability less easily absorbed, especially by young children. The physiological effect of the alterations in the phosphorus is not certain ; but as cows' milk is poor in organic phosphorus bodies, and these seem very needful to the human infant, heating may be considered to act detrimentally in decomposing these compounds.

The third group of changes, the least easily detected of all, are those which concern the "vital" properties of milk. Enzymes, agglutinins and precipitins, bodies presumably of value to the infant, are destroyed, and phagocytosis by the milk cells abolished. The so-called bactericidal power of fresh milk, which is really more inhibitory than bactericidal, is also destroyed. This is a great loss from the point of view of infant-feeding. Finally, clinical opinion, especially on the Continent, is steadily coming to the view that clean fresh milk is better for infants than heated milk. Though infantile scurvy is rare, other disturbances—such as anæmia, constipation and pyelitis—are more frequently met with in children brought up entirely on sterilised milk.

